# The American National Red Cross

**Published:** 1907-12

**Authors:** 


### The American National Red Cross

**Letter from Surgeon-General O'Reilly, U. S. Army — As member of the Executive Committee, American National Red Cross, sends resolutions adopted by the Red Cross relating to the commercial and other uses of the insignia.**

SIR—As a member of the Executive Committee of the American National Red Cross, I have been requested to forward to you the enclosed resolutions with the hope that you may find it expedient to kindly print them in your publication and lend what assistance you can in discharging the international duty assumed by this country in agreement with all the great powers, of protecting the Red Cross name and insignia from any use save that agreed upon by the treaty of Geneva.

In cases where the name and insignia are used as trade-marks, the suggestion would be of value that their use should be gradually eliminated within the next three or four years, in conformity with the provisions of the treaty that the prohibition of the use of the emblem and name should be enforced not later than five years after the treaty went into effect.

R. M. O'REILLY,

Surgeon General, U.S. Army, Member of the Executive Committee, the American National Red Cross.

National Headquarters, A.N.R.C.,
Washington, D.C., November 9, 1907.

Resolutions adopted by the Executive Committee of the American National Red Cross, October 18, 1907:

WHEREAS, By international agreement in the Treaty of Geneva, 1864, and the revised Treaty of Geneva, 1906, "the emblem of the Red Cross on a white ground and the words Red Cross or Geneva Cross" were adopted to designate the personnel protected by this Convention, and

WHEREAS, The Treaty further provides (Article 23) that "the emblem of the Red Cross on a white ground and the words Red Cross or Geneva Cross can only be used whether in time of peace or war, to protect or designate sanitary formations and establishments, the personnel and material protected by this Convention," and

WHEREAS, The American National Red Cross comes under the regulations of this Treaty according to Article 10, "volunteer aid societies, duly recognised and authorised by their respective Governments," such recognition and authority having been conferred upon the American National Red Cross in the Charter granted by Congress, January 5, 1905, Sec. 2, "The corporation hereby created is designated as the organisation which is authorised to act in matters of relief under said Treaty," and, furthermore,

WHEREAS, In the Revised Treaty of Geneva, 1906, in Article 27, it is provided that "the signatory powers whose legislation should not now be adequate, engage to take or recommend to their legislatures such measures as may be necessary to prevent the use by private persons or by societies other than those upon which this Convention confers the right thereto of the emblem or name of the Red Cross or Geneva Cross,"

*Be it Resolved,* That the Executive Committee of the American National Red Cross requests that all hospitals, health departments and like institutions kindly desist from the use of the Red Cross created for the special purpose mentioned above, and suggests that for it should be substituted some other insignia, such as a green St. Andrew's Cross on a white ground, to be named the "Hospital Cross", and used to designate all hospitals (save such as are under the Medical Departments of the Army and Navy and the authorised volunteer aid society of the Government), all health departments and like institutions, and, further,

*Be it Resolved,* That the Executive Committee of the American National Red Cross likewise requests that all individuals or business firms and corporations who employ the Geneva Red Cross for business purposes, kindly desist from such use, gradually withdrawing its employment and substituting some other distinguishing mark.

